# Ganoderma lucidum aqueous extract prevents hypobaric hypoxia induced memory deficit by modulating neurotransmission, neuroplasticity and maintaining redox homeostasis

**DOI:** 10.1038/s41598-020-65812-5

**Published:** 2020-06-02

**Authors:** Purva Sharma, Rajkumar Tulsawani

**Affiliations:** Defence Institute of Physiology and Allied Sciences (DIPAS), Lucknow Road, Timarpur, Delhi 110054 India

**Keywords:** Drug discovery, Neuroscience, Biomarkers, Diseases, Medical research, Neurology

## Abstract

Oxidative stress due to hypobaric hypoxia at extreme altitudes causes severe neuronal damage and irreversible cognitive loss. Owing to contraindications of current drug therapies, the aim of the study was to investigate memory enhancing potential of aqueous extract of *Ganoderma lucidum* (GLAQ) and underlying neuroprotective mechanism using rat hypobaric hypoxia test model. Rats exposed to hypobaric hypoxia showed deranged spatial memory in morris water maze test with hippocampal damage and vasogenic cerebral edema. All these changes were prevented with GLAQ treatment. Blood and biochemical analysis revealed activation of hypoxic ventilatory response, red blood cells induction, reversal of electrolyte and redox imbalance, and restoration of cellular bioenergetic losses in GLAQ treated animals. Notably, GLAQ treatment ameliorated levels of neurotransmitters (catecholamines, serotonin, glutamate), prevented glucocorticoid and α-synuclein surge, improved neuroplasticity by upregulating CREB/p-CREB/BDNF expression via ERK1/ERK2 induction. Further, restoration of nuclear factor erythroid 2-related factor with stabilization of hypoxia inducible factors and inflammatory markers were evidenced in GLAQ treated rats which was additionally established in gene reporter array using an alternative HT22 cell test model. Conclusively, our studies provide novel insights into systemic to molecular level protective mechanism by GLAQ in combating hypobaric hypoxia induced oxidative stress and memory impairment.

## Introduction

Chronic exposure to high altitude (HA) is incapacitating at both physiological and psychological level^1,2^. Ascent to extreme altitudes is life threatening due to maladies such as acute mountain sickness, severe hypoxia, and high altitude cerebral edema^3,4^. HA exposure leads to cognitive functional impairments in learning-memory, visual-verbal abilities, and decision making^5,6^. Cognitive losses exacerbate with increasing altitudes^7^. Brain with high basal oxygen demand is more prone to low partial pressure of oxygen, a condition known as hypobaric hypoxia (HH). Neuronal damage in hippocampus during oxidative stress is primarily due to accumulation of free radical and reactive oxygen species, which results in cellular bioenergetic failure, inflammation, blood brain barrier dysfunction and eventually neuronal cell death^8^. Despite the knowledge of severity, the only treatment currently available either reduces mortality or sequelae in mild cases. Nonspecificity of available drugs such as acetazolamide and dexamethasone have several contraindications and risk of anaphylaxis^9^. Wang *et al*., 2013. further reported cognitive impairment with the use of acetazolamide during HA exposure^10^. Moreover, failure to acclimatization with ascent of altitude is another serious innuendo that needs to be addressed.

**Figure 1:**
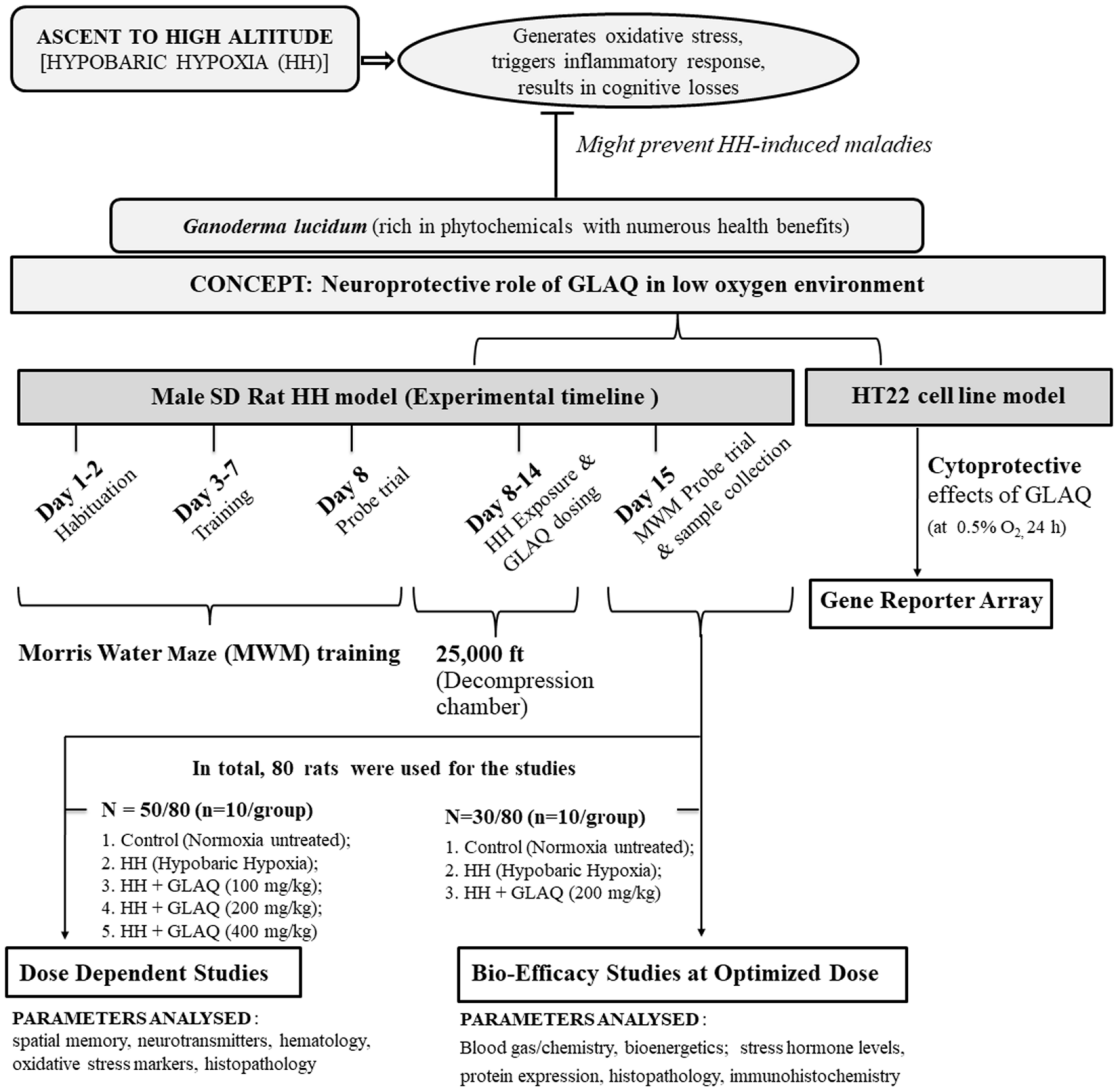
Schematic representation of study hypothesis and experimental design. *Ganoderma lucidum* widely used for its anti-oxidant and anti-inflammatory properties is hypothesised to prevent memory impairment under low oxygen environment. Rat hypobaric hypoxia (HH) model was used to study cognitive performance under stress in presence and absence of aqueous extract of *Ganoderma lucidum* (GLAQ). After training rats in Morris water maze, they were exposed to chronic HH at simulated altitude of 25,000 ft or 282 mmHg in decompression chamber for 7 days with simultaneous administration of GLAQ (0, 100, 200, 400 mg/kg body weight) every 24 h. After exposure, animals were sacrificed for study analysis to understand neuroprotective mechanism exhibited by GLAQ. Additional studies were conducted in hippocampal neuronal HT22 cells exposed to hypoxia (0.5% O_2_, 24 h) and treated with various concentrations of GLAQ (0, 25, 50, 100 µg/ml) using gene reporter array to delineate hypoxia responsive transcription factors.

In view of this, rapid acclimatization with use of herbal agents could be an effective strategy to withstand HH-induced cognitive deficit. Recently, various evidences surfaced supporting herbal supplementation modulating neurogenesis and protecting hippocampal functions such as memory^11,12^. *Ganoderma lucidum (G. lucidum)*, a medicinal mushroom known to synthesize phytochemicals such as polysaccharides, adenosine, ergosterols, ganoderic acids, coumarin, and triterpenoids is widely used for its anti-oxidant, anti-inflammatory, and immunomodulatory properties^13,14^. In particular, *G. lucidum* exhibits a broad spectrum of therapeutic properties with neuroprotective effects in numerous pathological conditions^15–17^. Previous studies from our group suggested that GLAQ exhibit anti-stress effects against HH via counteracting oxidative stress^18^ and the administration of extract for 90 days sub-chronically did not alter mean body weights, organ to body weight ratio, hematological or clinical markers *per se* up to 1000 mg/kg dose^19^. However, present study was carried out in intent to fill the lacunae behind neuroprotective efficacy of GLAQ against HH from systemic to molecular level as hypothesized in Fig. 1.

The experiments were conducted in male Sprague Dawley rats exposed to chronic hypobaric hypoxia (7 days at 25,000 ft or 282 mmHg) with simultaneous administration of GLAQ (0, 100, 200, 400 mg/kg body weight) every 24 h as depicted in Fig. 1. Spatial acquisition and memory retention were measured post exposure in neurobehavioral test and extent of brain damage was measured with hematoxylin and eosin (H&E) and cresyl violet (CV) staining. Oxidative stress was measured with malondialdehyde (MDA), reduced glutathione (GSH), total antioxidant capacity (TAC) and ratio of nicotinamide-adenine dinucleotide (NAD)/reduced NAD (NADH). Bioenergetic status (ATP, glucose, blood urea nitrogen, creatinine, lactate), blood gas/electrolyte/metabolite analysis was performed to envisage overall impact of stress. Further, cognitive dysfunctions have been linked with alteration in neurotransmitter synthesis/secretion, glutamate cytotoxicity and high levels of corticosterone with reports of hypoxia and inflammation severely effecting synaptic signaling in central nervous system^20–22^. We thus, investigated influence of HH on markers for neurotransmission, neurotoxicity, synaptic plasticity, anti-oxidant, and anti-inflammatory potential. Additionally, reporter gene array was employed to delineate the transcription factors specifically responding to hypoxic stress using murine hippocampal HT22 neuronal cell line exposed to hypoxia (0.5% O_2_, 24 h) in presence and absence of extract. The findings from this study demonstrate neuroprotective mechanism of aqueous extract of *Ganoderma lucidum* strongly suggesting that GLAQ is a promising therapy to overcome HA based cognitive disorders.

## Results

### GLAQ restores HH-induced memory deficit

**Figure 2:**
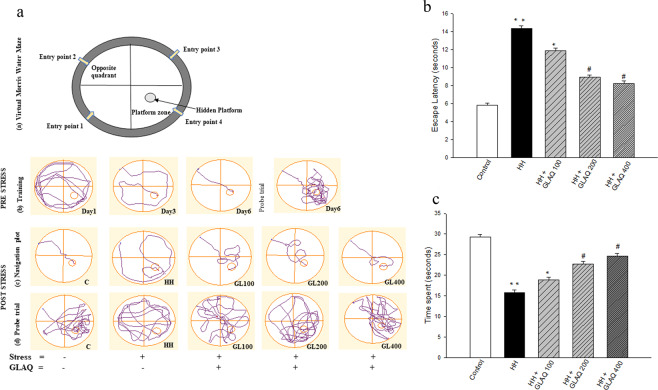
Protective effects of GLAQ on hypobaric hypoxia induced memory deficit. (**a**) shows the (a) schematic representation of virtual Morris Water Maze, (b) representative recorded track plot during training period (with platform) along with probe trial (without platform) before stress, (c) representative navigation plot for calculating escape latency and (d) track plot during probe trial to calculate time spent in platform zone post stress of control rats, exposed rats untreated and treated with GLAQ at doses 100 mg/kg (GL100), 200 mg/kg (GL200) and 400 mg/kg (GL400). (**b**,**c**) shows dose dependent protective effects of extract on escape latency (seconds), time spent in platform zone for determining spatial memory acquisition and reference memory respectively. Bars represent mean ± SEM (n = 10) with statistical significance level of *p < 0.05, **p < 0.01, *vs control and ^#^p < 0.05 vs HH.

From Morris Water Maze (MWM) training data (Fig. 2a,b), it was seen that control rats did not show memory variation after 7-day period when compared with pre-exposed rats. Post hoc analysis revealed that stress exposure significantly increased the escape latency (Fig. 2b) and decreased the time spent in platform zone during probe trial (Fig. 2c) as evident from the respective representative track plots (Fig. 2a-c,d). However, GLAQ administration showed dose dependent retention of memory thus indicating prevention of adverse effects of HH. Although the memory loss was reverted at 100 mg/kg dose, significant recovery was obtained at 200 mg/kg and 400 mg/kg doses with optimal dose effect obtained at 200 mg/kg.

### GLAQ regulates neurotransmitter levels at tissue and circulatory level during HH

**Figure 3:**
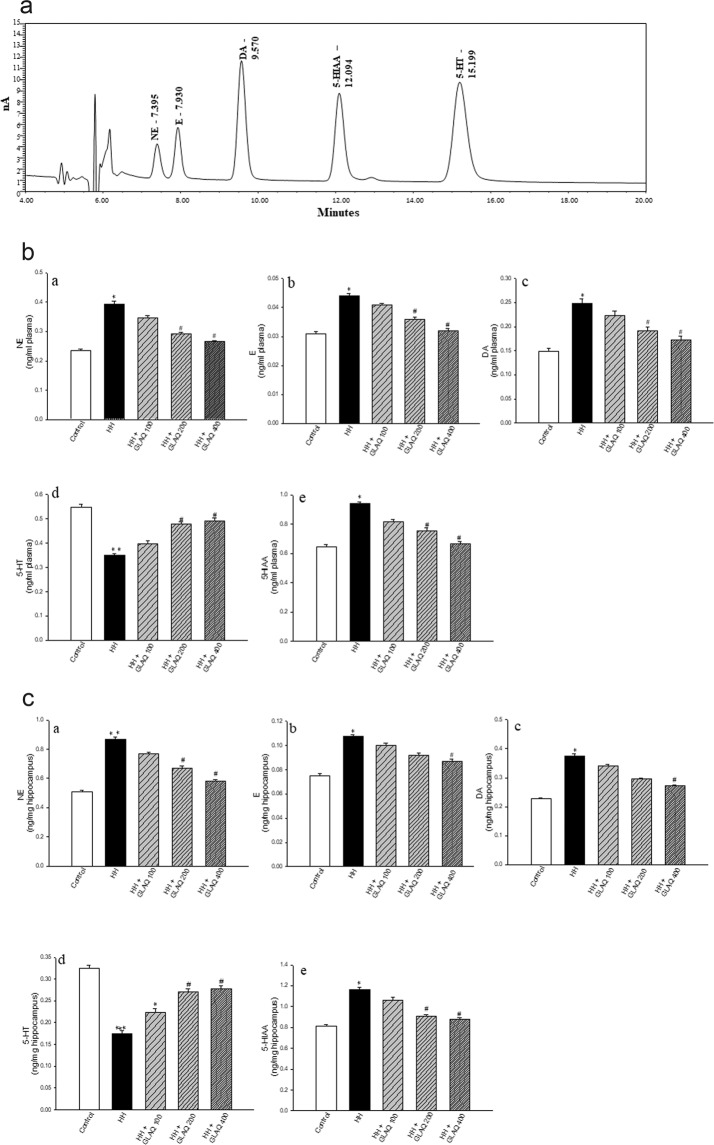
GLAQ regulates neurotransmitter levels at systemic and tissue level during hypobaric hypoxia. (**a**) shows RP-HPLC chromatogram of resolved standard peaks of nor epinephrine (NE), epinephrine (**e**), dopamine (DA), 5-hydroxytryptamine (5-HT), 5-hydroxyindoleacetic acid (5-HIAA) in standard mix. Bar graphs represents dose dependent protective effects of extract on differential levels of biogenic amines in systemic circulation or plasma (**b**-(a–e)) and hippocampus (**c**-(a–e)) on exposure to hypobaric hypoxia. Bar represents mean ± SEM (n = 6 hippocampus, n = 10 plasma), *p < 0.05, **p < 0.01, ***p < 0.001, *vs control and ^#^p < 0.05 vs HH.

Figure 3a depicts revere phase HPLC chromatogram of resolved standard peaks in standard mix. Catecholamines including nor-epinephrine (NE), epinephrine (E) and dopamine (DA), markers for fear response and stress, were significantly elevated in plasma (Fig. 3b,a–c) and hippocampus (Fig. 3c,a–c) post stress. Post exposure, serotonin or 5-hydroxytryptamine (5-HT) was reduced whereas its metabolite 5-hydroxyindole acetic acid (5-HIAA) was over expressed in plasma (Fig. 3b;d,e) and hippocampus (Fig. 3c;d,e) of HH group. Extract treatment prevented these anomalies in dose dependent manner at both systemic and tissue level with significant difference at doses of 200 mg/kg and 400 mg/kg. Intergroup variations between these two doses were not statistically significant which suggests that the optimal effective dose is 200 mg/kg. In other study, HH exposure increased glutamate levels in hippocampus and plasma (Table 1) while extract administration repressed the buildup.

**Table 1:** Protective efficacy of GLAQ on bio-energetic status and glucocorticoid release during hypobaric hypoxia.

Parameters	Control	HH	HH + GLAQ200	p-value
**Hippocampus**				
ATP (µM)	6.53 ± 0.38	4.30 ± 0.39*	8.11 ± 0.26^*#^	<0.0001
NAD/NADH	0.89 ± 0.07	0.61 ± 0.04*	1.08 ± 0.06^#^	<0.0001
TAC (µM)	222.5 ± 7.51	171.2 ± 9.91*	216.7 ± 9.58^#^	0.007
Glutamate (µM)	137.5 ± 5.50	155.8 ± 3.92*	149.7 ± 4.60	0.0449
Cortisol (pg/100 mg)	243.1 ± 10.9	412.5 ± 11.9*	316.4 ± 14.9^#^	<0.0001
Corticosterone(pg/100 mg)	106.2 ± 6.93	121.6 ± 7.21*	106.8 ± 7.60	0.0427
**Plasma**				
TAC (µM)	360.0 ± 10.1	219.8 ± 11.2*	377.1 ± 10.3^#^	<0.0001
Glutamate (µM)	91.9 ± 6.60	137.8 ± 4.54*	114.6 ± 2.81^*#^	<0.0001
Cortisol (ng/ml)	5.20 ± 0.20	6.24 ± 0.23*	5.98 ± 0.21^#^	0.009
Corticosterone (ng/ml)	51.9 ± 3.63	75.7 ± 4.71*	56.3 ± 4.65^#^	0.0024

Values are expressed as mean ± SEM (n = 6 hippocampus, n = 10 plasma).

*p < 0.05 vs control and ^#^p < 0.05 vs HH.

### GLAQ prevents HH-induced vasogenic cerebral edema and neuronal damage

**Figure 4:**
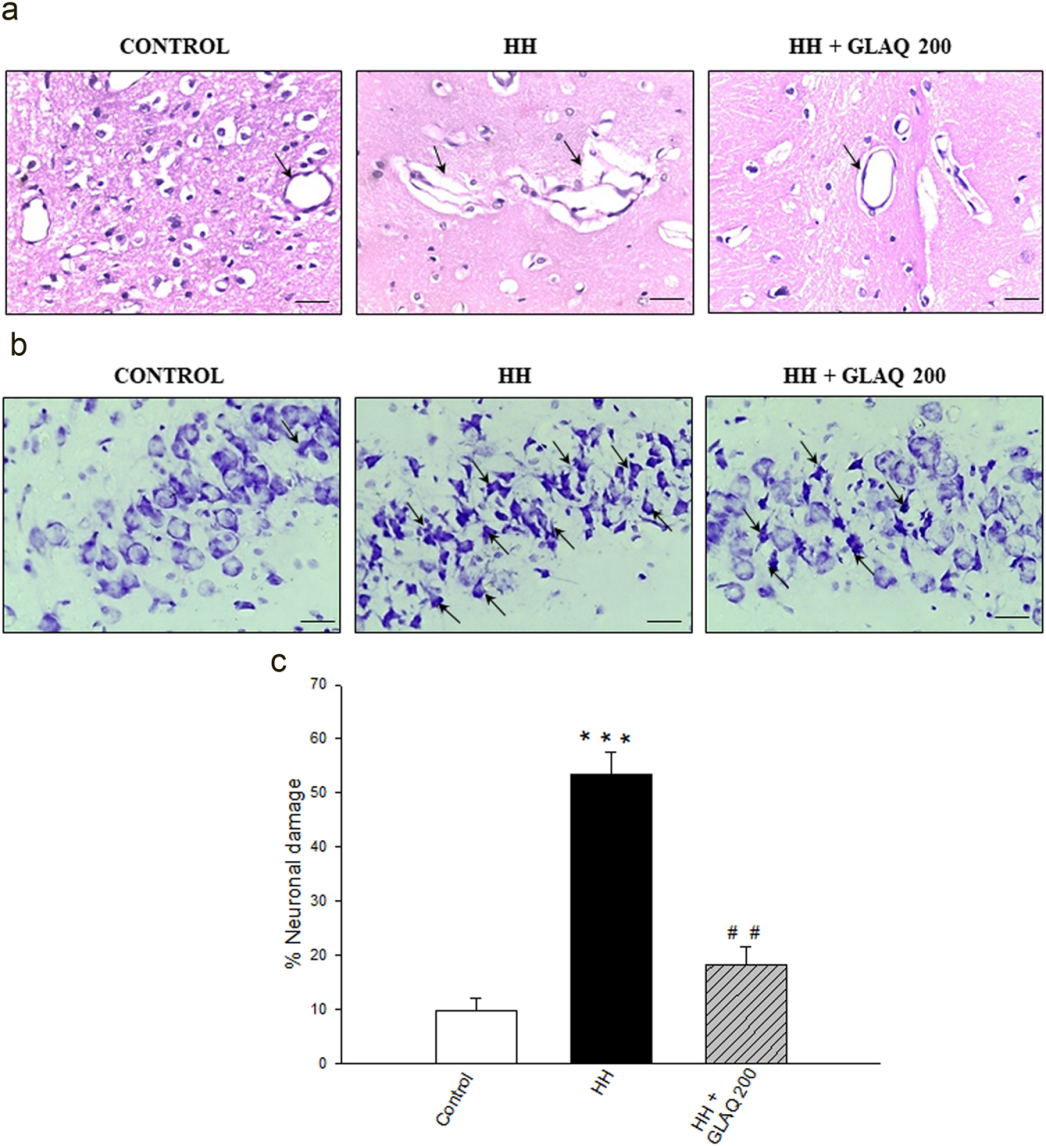
GLAQ prevents hypobaric hypoxia-induced vasogenic cerebral edema and neuronal damage. The representative photomicrographs of brain tissue: (**a**) H&E stained coronal sections from cortical region of control and exposed rats in presence and absence of extract. The widened peri-vascular spaces (as indicated with arrows), deformation of vascular parameter in HH group is notably reduced in GLAQ supplemented animals at 200 mg/kg dose. (**b**) CV stained coronal sections of brain shows hippocampal region of control and exposed rats in presence and absence of extract. Post stress, the hippocampal neurons in HH group exhibited cell shrinkage and nuclear pyknosis as indicated with arrows evidencing neuronal damage which is evaluated as (**c**) percentage neuronal damage. The damage was remarkably controlled in presence of GLAQ at 200 mg/kg dose. Bars are mean ± SEM (n = 4), ***p < 0.001 vs control and ^##^p < 0.01 vs HH. Magnification 400×, scale bar 200 µm.

Enlarged perivascular spaces or Virchow-Robin spaces (VRS) due to fluid accumulation is a characteristic of vasogenic cerebral edema^23^. In H&E staining, clearly defined edema was observed in HH group (as indicated with arrows) when compared with unexposed control whereas extract treatment improved the structural integrity of the vasculature (Fig. 4a). On other hand, CV staining revealed densely stained neurons exhibiting cellular shrinkage evidencing pyknosis (Fig. 4b; as indicated with arrows) and HH-induced neuronal damage was remarkably reduced in GLAQ treated animals (Fig. 4c).

### GLAQ facilitates acclimatization by activating Hypoxic Ventilatory Response (HVR) during HH

With no significant changes in white blood cells (WBC) count, an increase was observed in red blood cells (RBC), hematocrit (Hct) and hemoglobin (Hb) levels of HH group with additional increase in later three parameters in presence of extract (Table 2). Table 3 depicts protective efficacy of GLAQ on blood gas, electrolytes and metabolites in exposed animals. HH exposure caused oxygen deficit which was reflected by remarkably low partial pressure of oxygen (pO_2_) and oxygen saturation (sO_2_). Further, the acidic pH indicates respiratory acidosis leading to increased partial pressure of carbon dioxide (pCO_2_) which resulted in metabolic acidosis thereby decreasing the HCO_3_ concentration. Base excess (BE) deficit in comparison to control also supports prevalent acidosis. Increased blood lactate (lac) indicated fatigue in HH group. An increase in levels of sodium (Na), potassium (K) and chloride (Cl), blood urea nitrogen (BUN)/urea, creatinine and decrease in ionized calcium (iCa), glucose, anion gap (AnGAP) was observed in HH group. Extract treatment significantly improved pO_2_ and sO_2_ levels. The blood pH shifted towards the alkaline, but within the physiological range (7.35–7.45) with GLAQ treatment. pCO_2_, total carbon dioxide (TCO_2_) levels and lac were reduced along with restoration of BE in presence of extract. Extract treatment facilitated amelioration of altered levels of blood-electrolytes (Na, K, Cl, iCa) and metabolites (glucose, BUN/urea, creatinine, AnGAP) in exposed treated group.

**Table 2:** Protective efficacy of GLAQ on oxidative stress (MDA, GSH) and hematological markers (WBC, RBC, Hct, Hb) against hypobaric hypoxia.

Parameters	Control	HH	HH + GLAQ100	HH + GLAQ200	HH + GLAQ400	p-value
**Plasma**						
MDA (µM)	670.5 ± 34.1	783.3 ± 39.2*	722.8 ± 29.8	704.5 ± 31.2^#^	677.7 ± 33.6^#^	0.0455
GSH (nM)	1.14 ± 0.06	0.75 ± 0.04*	0.89 ± 0.05	1.06 ± 0.06^#^	1.11 ± 0.05^#^	<0.0001
**Blood**						
WBC (10^3^/mm3)	5.54 ± 0.11	5.52 ± 0.15	5.51 ± 0.16	5.49 ± 0.17	5.50 ± 0.17	0.997
RBC (10^6^/mm3)	7.40 ± 0.28	9.43 ± 0.19*	9.45 ± 0.26*	9.47 ± 0.18*	10.3 ± 0.25*	<0.0001
Hct (%)	47.0 ± 1.52	55.8 ± 0.64*	55.9 ± 0.63*	56.2 ± 0.54*	56.4 ± 0.90*	<0.0001
Hb (g/dl)	14.0 ± 0.42	21.6 ± 0.23*	21.7 ± 0.20*	22.8 ± 0.24*	22.9 ± 0.27*	<0.0001

Values are expressed as mean ± SEM (n = 10). *p < 0.05 vs control and ^#^p < 0.05 vs HH.

**Table 3:** Protective efficacy of GLAQ on blood gas/electrolytes/metabolites against hypobaric hypoxia.

Parameters (BLOOD)	Control	HH	HH + GLAQ200	p-value
**Blood Gas**				
pH	7.38 ± 0.01	7.26 ± 0.03*	7.37 ± 0.02^#^	0.0008
pCO_2_ (mmHg)	45.3 ± 0.70	48.9 ± 1.49	43.4 ± 1.88^#^	0.0365
pO_2_ (mmHg)	90.7 ± 1.55	61.2 ± 1.22*	81.4 ± 1.83*^#^	<0.0001
BE (mmol/L)	1.20 ± 0.42	−4.30 ± 0.30*	−0.50 ± 0.53*^#^	<0.0001
HCO_3_ (mmol/L)	26.5 ± 0.42	21.1 ± 0.87*	24.2 ± 0.67^#^	<0.0001
TCO_2_ (mmol/L)	26.9 ± 0.52	23.6 ± 0.45*	25.6 ± 0.67	0.0011
sO_2_ (%)	91.8 ± 2.35	56.4 ± 1.05*	89.0 ± 4.40^#^	<0.0001
Lac (mmol/L)	2.61 ± 0.17	6.38 ± 0.95*	4.02 ± 0.43^#^	0.0007
**Blood Electrolytes/Metabolites**				
Na (mmol/L)	140.0 ± 0.49	143.6 ± 0.81*	141.6 ± 0.60	0.0021
K (mmol/L)	5.01 ± 0.12	6.18 ± 0.10*	5.50 ± 0.10	<0.0001
Cl (mmol/L)	103.7 ± 1.09	112.6 ± 1.20*	108.5 ± 1.26	<0.0001
iCa (mmol/L)	1.26 ± 0.02	1.18 ± 0.03	1.27 ± 0.02	0.0276
Glucose (mg/dl)	136.1 ± 7.40	79.7 ± 4.35*	102.3 ± 6.89^#^	<0.0001
BUN/Urea (mg/dl)	20.4 ± 1.55	25.0 ± 1.54*	19.4 ± 0.72^#^	0.0137
Creatinine (mg/dl)	0.39 ± 0.01	0.43 ± 0.01*	0.40 ± 0.01	0.0233
AnGAP (mmol/L)	14.9 ± 0.50	16.5 ± 0.82	15.4 ± 0.45	0.2530

Values are expressed as mean ± SEM (n = 10). *p < 0.05 vs control and ^#^p < 0.05 vs HH.

### GLAQ prevents HH-induced oxidative stress, energy deficit and glucocorticoid toxicity

Experimental animals exposed to HH exhibited increase in plasma MDA levels which was concomitant with decreased plasma GSH levels. Extract treatment dose dependently ameliorated changes in levels of plasma MDA and GSH and effects were appreciable at 200 mg/kg and 400 mg/kg doses (Table 2). Similarly, rats exposed to HH revealed compromised plasma and hippocampal TAC levels which were restored with extract treatment (Table 1). Further, HH exposure repressed the intracellular levels of ATP and NAD/NADH in hippocampus which were restored and further augmented with GLAQ administration (Table 1). Glucocorticoids namely, cortisol and corticosterone were found to be elevated in both hippocampus and plasma of HH group (Table 1). However, presence of extract restrained glucocorticoid buildup which might prevent the neurotoxicity associated with it.

### GLAQ regulates protein expression to prevent inflammation, oxidative stress and improves synaptic plasticity at tissue and systemic level during HH

**Figure 5:**
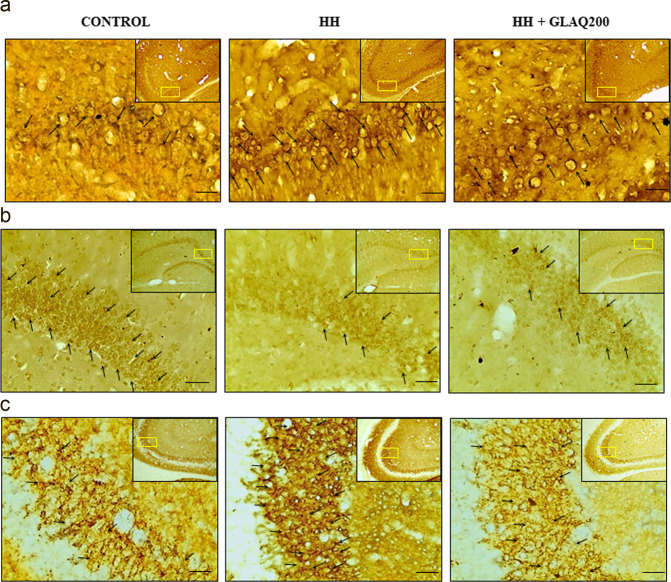
Immunohistochemical staining of hypobaric hypoxia exposed rat brain in presence and absence of extract. Representative photomicrographs of brain tissue shows positively stained pyramidal neurons (as indicated with arrows) for immune reactivity of (**a**) HIF1α in CA3 region, (**b**) BDNF in dentate gyrus region, (**c**) SYN1 in CA3 region of hippocampus in panels left to right as control, HH and HH + GLAQ200 group at 400X magnification and scale bar 200 µm (with reference of 100X sections on top right with defined yellow rectangular area for 400×). Presence of extract restored BDNF expression whereas suppressed the HH-induced overexpression of HIF1α and SYN1.

Table 4 depicts regulation of HH-induced protein markers of plasma and hippocampus in presence and absence of extract. Hypoxia inducible factor 1α (HIF1α) showed increased expression post stress which was dampened by the presence of extract. Concomitant observation was seen in qualitative immunohistochemistry (IHC) results (Fig. 5a), where the number of HIF 1α positive neuronal cells (indicated with arrows) in HH group outnumbered the ones in control, which were reduced in presence of extract. Also, HIF 2α and vascular endothelial growth factor (VEGF) expression increased in HH group which were repressed in GLAQ treated group. Presence of GLAQ also suppressed level of inflammatory cytokine, nuclear factor kappa-light-chain-enhancer of activated B cells (NFĸB) which was overexpressed in HH group. Expressions of nuclear factor erythroid 2-related factor 2 (NRF2) and its downstream target heme oxygenase 1 (HO1) decreased in HH group which were restored in presence of extract. Further, increased levels of erythropoietin (EPO) following hypobaric hypoxia were augmented with extract treatment.

**Table 4:** GLAQ regulates hypobaric hypoxia induced multiple protein pathways associated with oxidative homeostasis and neuroplasticity.

Markers	PLASMA				HIPPOCAMPUS			
	Control	HH	HH + GLAQ200	p-value	Control	HH	HH + GLAQ200	p-value
**Hypoxia and Oxidative Stress**								
HIF1α (pg/ml)	473.6 ± 6.95	557.1 ± 4.12^*^	498.6 ± 5.12^*^	<0.0001	494.7 ± 8.63	564.9 ± 7.98^*^	504.3 ± 7.21^#^	<0.0001
HIF2α (pg/ml)	281.8 ± 7.80	327.1 ± 7.95^*^	251.6 ± 7.71^*#^	<0.0001	200.5 ± 9.85	283.4 ± 8.43^*^	221.3 ± 7.65^#^	<0.0001
VEGF (pg/ml)	186.9 ± 9.98	260.6 ± 8.85^*^	214.5 ± 8.52^#^	<0.0001	200.9 ± 5.73	265.4 ± 6.50^*^	198.4 ± 5.41^#^	<0.0001
NFĸB (ng/l)	180.9 ± 7.82	280.6 ± 9.25^*^	222.9 ± 10.9^*#^	<0.0001	204.8 ± 2.44	271.6 ± 2.01^*^	204.9 ± 2.73^#^	<0.0001
NRF2 (pg/ml)	543.5 ± 3.90	448.2 ± 5.21^*^	528.8 ± 7.81^#^	<0.0001	425.9 ± 4.54	311.9 ± 5.95^*^	394.5 ± 4.90^*#^	<0.0001
HO1 (ng/ml)	87.3 ± 1.76	64.2 ± 1.23^*^	76.8 ± 1.11^*#^	<0.0001	68.3 ± 2.28	52.2 ± 2.49^*^	62.9 ± 2.31^#^	0.0008
EPO (pg/ml)	221.9 ± 5.83	281.7 ± 4.26^*^	299.4 ± 5.20^*^	<0.0001	125.5 ± 5.81	169.7 ± 6.62^*^	185.9 ± 5.16^*^	<0.0001
**Neuroplasticity**								
CREB (ng/ml)	1.78 ± 0.05	1.07 ± 0.04^*^	1.56 ± 0.06^*^	<0.0001	1.65 ± 0.03	1.42 ± 0.04^*^	1.53 ± 0.09	0.0479
p-CREB (pg/ml)	20.6 ± 0.50	16.2 ± 0.71^*^	18.9 ± 0.60^#^	<0.0001	23.4 ± 0.94	19.6 ± 0.67^*^	21.3 ± 0.48	0.0073
ERK1/2 (pg/ml)	808.9 ± 23.4	555.8 ± 21.5^*^	667.7 ± 27.2^*#^	<0.0001	410.3 ± 9.61	323.7 ± 9.92^*^	382.8 ± 8.97^#^	<0.0001
BDNF (ng/l)	39.2 ± 1.32	26.8 ± 1.29^*^	32.6 ± 1.78^*#^	<0.0001	47.6 ± 1.42	22.2 ± 1.01^*^	33.6 ± 1.48^*#^	<0.0001
SYN1(pg/ml)	240.8 ± 10.2	355.3 ± 9.47^*^	292.3 ± 8.97^*#^	<0.0001	159.9 ± 3.46	177.6 ± 5.43^*^	168.9 ± 5.28	0.0486
α SYN (pg/ml)	44.1 ± 1.71	63.4 ± 1.63^*^	55.6 ± 1.80^*#^	<0.0001	58.2 ± 2.11	79.4 ± 1.37^*^	61.9 ± 1.36^#^	<0.0001

Values are expressed as mean ± SEM (n = 6 hippocampus, n = 10 plasma). *p < 0.05 vs control and ^#^p < 0.05 vs HH.

Cyclic AMP response element-binding protein (CREB) phosphorylation in hippocampus has been associated with spatial memory^24,25^. In the present study, levels of CREB and phospho-CREB (p-CREB) were decreased in circulation and hippocampus and restored with extract treatment. Extracellular signal-regulate kinases (ERK1/ERK2) expression was reduced in HH group and normalized to control values in presence of extract. Brain-derived neurotrophic factor (BDNF), a downstream target of CREB was also decreased in HH group and restored with extract. IHC confirmed the same, as number of BDNF positively stained neuronal cells (indicated with arrows) declined in HH group and were more numerous in presence of extract (Fig. 5b). Additionally, levels of synapsin 1 (SYN 1) increased in HH group and were reduced with extract treatment. This finding was confirmed qualitatively with IHC staining (Fig. 5c). Further, overexpression of alpha synuclein (α SYN) in plasma and hippocampus in HH group was suppressed by the extract.

### *In-vitro* study: Cytoprotective mechanism at transcriptional level by GLAQ in hypoxia exposed HT22 cells

**Figure 6:**
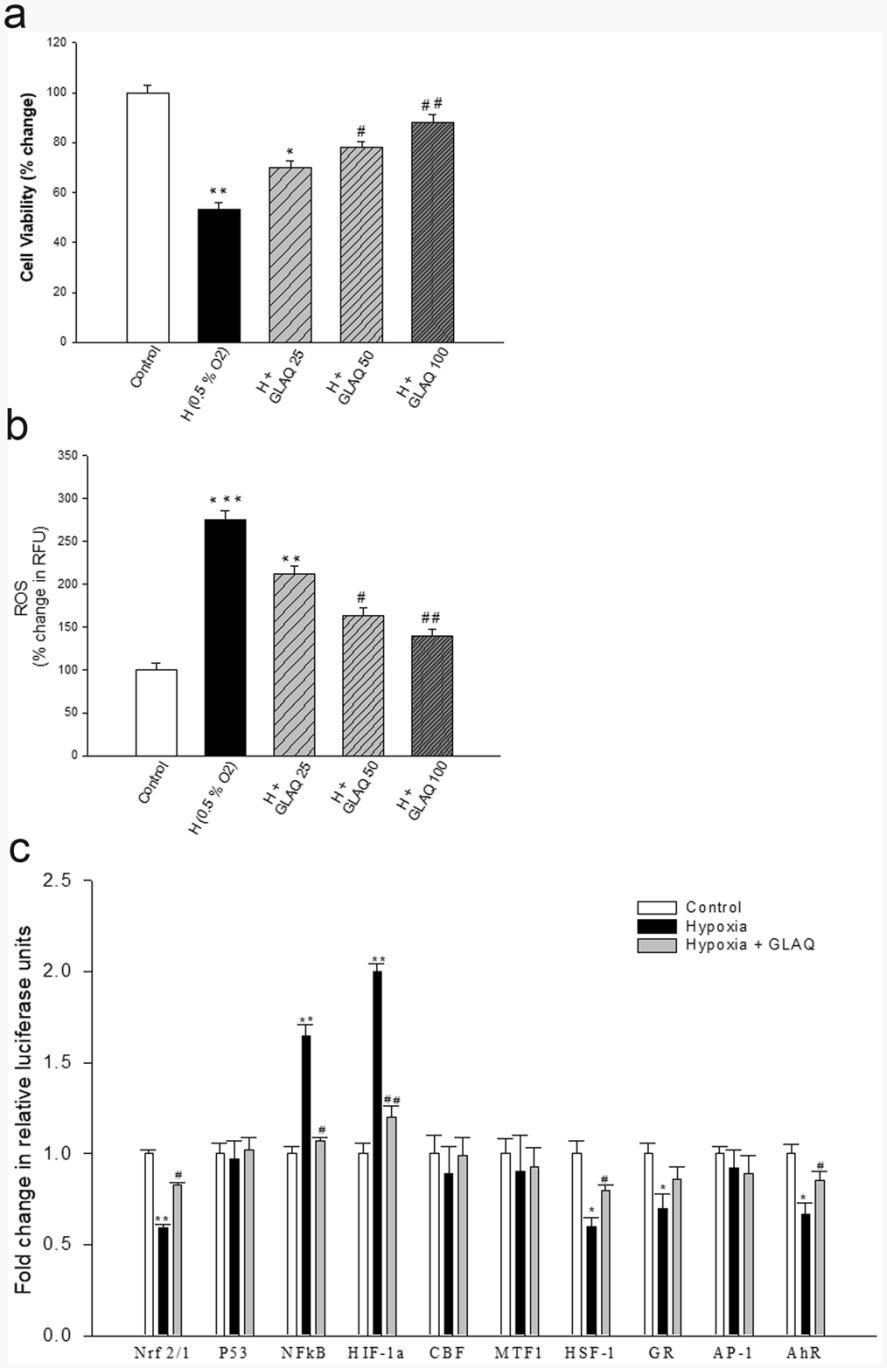
Cytoprotective efficacy of GLAQ in hypoxia exposed HT22 cells. Dose dependent protective effects of extract on hypoxia induced (**a**) cell death with MTT assay [Basal values OD_570_ = 0.94 ± 0.01 (normoxic control)] and (**b**) % change in fluorescence of ROS generation detected with DCFDA dye [Basal values RFU_485Ex/530Em_ = 91736 ± 1086 (normoxic control)] during hypoxia where each bar represents mean ± SEM (n = 8). (**c**) represents fold change in expression of transcription factors in gene finder reporter array where six pathways have been found to be altered (up/down regulated) in response to hypoxia; each of which was ameliorated in presence of extract. Each bar represents mean ± SEM (n = 4). *p < 0.05, **p < 0.01 vs control and ^#^p < 0.05, ^##^p < 0.01 vs hypoxia (0.5% O_2_).

Figure 6 depicts the protective effects of GLAQ in HT22 cells exposed to hypoxia. The viability of untreated exposed cells reduced to 53% (Fig. 6a) with nearly three times accumulation of reactive oxygen species (ROS) (Fig. 6b). However, extract treatment dose dependently increased survivability and reduced ROS with maximal efficacy at 100 µg/ml dose. The data obtained from gene reporter array (Fig. 6c) indicated that hypoxia caused upregulation of transcriptional factors: HIF1α and NFĸB to 2-folds and 1.65 folds, respectively as compared to normoxia control. Further, NRF1/NRF2 expression was reduced to nearly half along with notable reduction in heat shock factor-1 (HSF1), glucocorticoid receptor (GR) and aryl hydrocarbon receptor (AhR). No significant changes were observed in expression of tumor protein 53 (p53), metal regulatory transcription factor 1 (MTF1), activator protein 1 (AP-1) and CCAAT- binding factor NF-Y (CBF/NF-Y). The presence of extract repressed the overexpression of HIF1α, NFĸB and restored expression of NRF2/NRF1, HSF1, GR and AhR. Conclusively, extract was able to regulate multiple signaling pathways especially HIF1α, NFĸB, NRF2/NRF1 during hypoxia which in turn may prevent a range of pathogenic responses in HT22 neuronal cells.

## Discussion

The current finding that GLAQ administration significantly improved the escape latency and time spent in platform zone in MWM test demonstrates its memory enhancing potential against HH-induced cognitive deficit. In line, recent reports have documented anti-amnesic effects of *G. lucidum* in promoting cognitive functions and neuronal progenitor proliferation^26,27^. In our preliminary studies, extract *per se* at 100 mg/kg did not reveal any significant changes between memory parameters of untreated normoxic and treated normoxic group (Supplementary Table TS1 and Figure FS1) negating individual effect of extract on memory under normal conditions. Further, administration of GLAQ at 100, 500 or 1000 mg/kg dose in experimental rats for 90 days did not produce any toxic or beneficial effects *per se* in absence of stressful environment^19^. The changes induced by GLAQ *per se* at 100 mg/kg, it’s five- and ten- times higher dose on organ-body weight index, hematological and clinical parameters were statistically non-significant, within normal range of untreated control and showed absence of adverse impact on histopathology of vital organs including brain^19^. The findings reveal that GLAQ *per se* is not effective however, exhibited protective response against multiple stressors including HH^19^ thus, providing basis for present dose dependent studies. Further, beneficial effects of quantified phytoconstituents of GLAQ such as coumarin and ganoderic acid A under low oxygen environment are not known and thus, can be speculated that an array of bioactive compounds present in GLAQ might be responsible for its protective efficacy for which further studies are required. CV staining revealed damage in long term potentiating pyramidal neurons in CA3 region of hippocampus due to pyknosis and cellular shrinkage post HH exposure. H&E staining showed severely deformed blood vessels and enlarged peri-vascular spaces or VRS in HH group with similar reports owing to HA induced brain damage^28^. VRS anomalies are also common in dementia, focal brain dysfunction^29^, multiple sclerosis^30^ which further corroborates the observed memory loss. However, presence of GLAQ not only reduced the neuronal damage but also maintained structural integrity of brain vasculature thus, substantiating improved cognitive performance of extract treated group. Elsewhere, polysaccharide extract from *G. lucidum* has been shown to reduce cerebral infarct area, neuronal apoptosis with protection of cortical neurons during ischemia and hypoxia/reoxygenation injury^31^. HA induced oxidative stress is involved in etiology of numerous neurodegenerative disorders^32,33^. HH exposure indeed suppressed total anti-oxidant capacity and GSH levels thereby triggering the membrane peroxidation reflected in MDA buildup. However, extract administration dose dependently alleviated HH-induced oxidative stress in rats implying that, anti-oxidant and neuroprotective properties of GLAQ are inter-linked.

Neurotransmitters are an important class of neural markers associated with memory and disturbance in oxygen homeostasis affects neurotransmitter synthesis causing impaired neuronal functions^20^. The expression pattern of biogenic amines post hypoxia exposure obtained from RP HPLC analysis in present study are in line with previous reports^20,34^. Of note, while NE, E and 5-HT contents were consistent with previous studies^34,35^, the DA basal content was considerably high as compared to reported levels^36^ for which conceivable confounding factor in methodological complications could not be eliminated. It is known that hypoxia downregulates the expression of tryptophan hydroxylase, a rate limiting enzyme dependent on oxygen which is responsible for 5-HT synthesis^37^ and 5-HT levels were significantly reduced along with elevated levels of 5-HIAA in HH group. However, further study needs to be done to validate molecular targets involved in neurotransmitter release. Besides, glutamate and glucocorticoids were alarmingly high in plasma and hippocampus post stress. Surge in hippocampal and circulatory levels of endogenous/exogenous glucocorticoids is frequently associated with cognitive impairment, more so, steroidal dementia is known causal effect of glucocorticoid neurotoxicity^21,38^. However, presence of extract ameliorated levels of neurotransmitters, and prevented HH-induced glutamate and glucocorticoid flux at both systemic and tissue level.

Acclimatization in presence of extract at induced altitude was examined with blood- gas/electrolytes/metabolites parameters. HH exposure critically impacted pO_2_ and SO_2_; reduced pH, HCO_3_, BE and increased pCO_2_, lac in HH group indicative of respiratory and metabolic acidosis, respectively^39^. The electrolyte imbalance in K, Na and Cl ion fluxes has been elsewhere related to impairment in kidney functioning^40^ justifying the anomalies in BUN, creatinine, and glucose levels in HH group. It is known that high altitude induced-HVR accelerates CO_2_ elimination and produces respiratory alkalosis which is crucial to acclimatization^41^. Interestingly, extract treated animals showed activated HVR with improvement in blood gas/chemistry. Further, HH exposure declined the levels of ATP and NAD/NADH which agrees with previous reports^42,43^. Extract administration not only restored their levels but surpassed control levels to possibly facilitate coping up with anaerobic respiration occurring during hypoxia exposure. Thus, GLAQ administration might help in better acclimatization via O_2_ homeostasis at systemic level during HH exposure.

Role of GLAQ in activation or deactivation of hypoxia-responsive molecular pathways was further elucidated. It is known that, oxidative stress in hypoxia triggers depression in synaptic transmission, neuroplasticity whilst prolonged exposure leads to neuroinflammation and neuronal death^44,45^. Survival and functional maintenance of neurons are dependent on neurotrophins like BDNF which plays an important role in brain development and synaptic plasticity^46^. BDNF is controlled by CREB transcription factor through its phosphorylation by multiple pathways including MAPK/ERK^47^, and disturbance in CREB phosphorylation leads to progressive neurodegeneration in hippocampus^48^. GLAQ treatment stimulated the expression of CREB, p-CREB via ERK1/ERK2 and restored BDNF expression in exposed rats. Previous studies have reported involvement of BDNF in neuroprotection under different stress stimuli by triterpenoid and polysaccharides extract obtained from *Ganoderma lucidum*^17,49,50^. More so, presence of extract suppressed the HH-induced over expression of both SYN1 and α-SYN, which are known to reflect synaptic distribution^51^. In particular, hypoxia-induced α-SYN aggregation has been shown to negatively impact neuronal survival^52^.

Diverse cellular adaptive responses are generated against oxidative insults including redox homeostasis, stress response, inflammation, angiogenesis etc. Hypoxia induced oxidative stress and inflammation often develop concurrently influencing cellular signaling pathways such as NFĸB/HIF^53,54^. Hypoxia alters neuronal cellular redox state and induces high levels of inflammatory cytokines such as NFkB^55^. Further, functional crosstalk between NRF2 and NFkB pathways under oxidative stress has been reported where, higher NRF2 levels decreases inflammation while its lower levels results in cytokine surge which is transcriptionally regulated by NFkB^56^. NRF2 has lately emerged as a therapeutic target in oxidative neurodegenerative pathological processes as it regulates cytoprotective genes^57^. It is reported that astrocytes expressing NRF2 protect neurons from oxidative stress whereas its knock out caused multiple sclerosis^58^ and of note, NRF2 activators have made their way in clinical trials against multiple sclerosis^59^. Thus, NRF2 is a critical target and its induction helps in cell survival. In the present study, it was observed that oxidative stress enhanced NFkB expression which further exerted immune response by repressing NRF2 and HO1 in both plasma and hippocampus. Elsewhere, it is reported that NRF2 is downregulated during hypoxia^60^ and the findings of the study highlight that oxidative stress might trigger the interplay between NFĸB and NRF2 at cellular and tissue levels. The GLAQ extract due to its high phenolic content^19^ and antioxidant activity possibly helped in reduction of oxidative stress thereby, decreasing neuronal inflammation observed in hippocampus of exposed treated group.

Interestingly, HIF2α, a tissue specific marker was over expressed post stress, in addition to HIF1α. HIF are heterodimeric transcription factors composed of O_2_-regulated α subunits (HIF1α or HIF2α), and a constitutively expressed ARNT/HIF1β subunit. HIF1α is primarily responsible for transcriptionally activating hypoxia related set of genes for regulating vascular growth, erythropoiesis, bioenergetic homeostasis, and cell survival^61,62^. Further, upregulation of VEGF in tissue along with leakage of inflammatory cytokines in plasma, causing either local or systemic inflammation, may account for the observed neuronal damage, perturbed vasculature and edema in HH group. However, presence of GLAQ counteracts hypoxia-induced disturbances in NFĸB/NRF2/HIF axis and expression of associated downstream targets. Moreover, GLAQ administration augmented EPO expression which explains the additional increase observed in RBC count, Hct and Hb levels evincing that GLAQ might regulate EPO release as an adaptive response to hypoxic insult by stimulating RBC production^63^.

**Figure 7:**
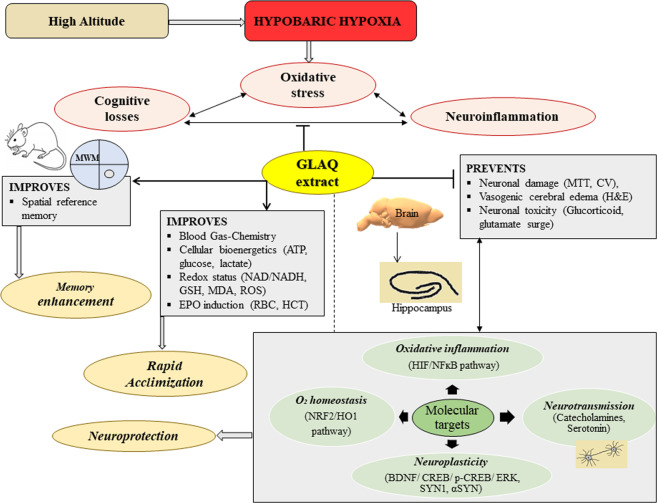
Schematic representation of neuroprotective mechanism of aqueous extract of *Ganoderma lucidum* during hypobaric hypoxia. Hypobaric hypoxia (HH) induced neurodegeneration under severe oxidative stress eventually leads to cognitive losses. Aqueous extract of *Ganoderma lucidum* (GLAQ) known for its anti-oxidant properties dose dependently improved spatial memory in exposed rats during morris water maze (MWM) test, indicating its memory enhancing potential. Further, GLAQ ameliorated perturbed blood gas/chemistry, prevented bioenergetic losses (ATP, glucose, lactate), and improved overall redox status (NAD/NADH, GSH, MDA) thus, countering the HH-induced oxidative stress and facilitating acclimatization. At cellular level, presence of extract alleviated glucocorticoid and glutamate buildup, modulated neurotransmission, and improved neuroplasticity by stimulating CREB/p-CREB/BDNF pathway via ERK1/ERK2 induction and reducing α-SYN and SYN1 surge. Induction of O_2_ homeostasis markers such as EPO (concomitant with increased levels of RBC) and NRF2/HO1 pathway along with stabilization of HIF1α/NFĸB axis in presence of GLAQ possibly reduce the neuronal inflammation thus reducing HH-induced neuronal damage and vasogenic cerebral edema.

Above mentioned discussions indicate that GLAQ controls hypoxia-induced pathophysiological disturbances through regulation of NFĸB/NRF2/HIF axis, atleast to certain extent. Thus, additional studies at cellular levels were conducted using HT22 hippocampal neuronal cell line. In particular, it been established as *in vitro* model for studies relevant to hippocampal dependent memory formation and dysfunction^64^. With relevance to hypoxia, brain tissue pO_2_ 1% or less fairly mimics *in vivo* hypobaric hypoxia equivalent to extreme altitudes such as 25,000 ft^65^. Further, within the brain, oxygen consumption is highly dynamic and region specific and hippocampal neurons are first ones to lose their electrical activity during hypoxia insult^66^. It is also reported that 0.5% O_2_ concentration is close to half maximal lethal concentration in HT22 neuronal cells^55^. In present study, it was observed that GLAQ exhibited cytoprotective effects in hypoxia exposed HT22 cells by scavenging accumulated ROS. Reporter array studies revealed reduced NRF2/NRF1 expression suggestive of compromised cellular anti-oxidative defense mechanism during hypoxia. Further, it is known that, HIF1α and AhR both compete for binding to ARNT thus exhibiting link between hypoxia and AhR-induced gene-expression profiles^67^. HT22 cells exposed to hypoxia revealed higher HIF1α expression with suppressed AhR which agrees to other reports^68^. It is also documented that in response to metabolic insult during renal ischemia both HIF and HSF1 were activated^69^. In present study, hypoxia resulted in metabolic stress however HSF1 expression was downregulated in HT22 cells. The unconventional regulatory link between oxygen-sensing and heat shock pathways have been reported elsewhere^70^. Further, glucocorticoids are known to modulate GR mediated gene expression by binding to glucocorticoid response element in the promoter region of target genes^71^, or an interference with other transcription factors such as NFĸB to inhibit their transcriptional activity^72,73^. This explains the antagonism between NFĸB and GR expression observed in our studies where, cells exposed to hypoxia showed higher NFĸB levels and decreased GR expressions. However, hypoxic cells treated with extract showed restored metabolism and reduced inflammation (NFĸB levels) suggesting relief from hypoxia-induced decrease in HSF1 and GR expressions. Hypoxia exposure to HT22 cells did not reveal any noticeable change in expressions of p53, MTF1, AP1 and CBF/NF-Y. Though this study in neuronal cells was designed to obtain information on role of GLAQ in regulation of NFĸB/NRF2/HIF axis as a proof of concept from previous studies, changes in expressions of HSF1 and GR was evident which opens new avenue for further investigations. Based on the current experimental findings, an overall protective mechanism of GLAQ against hypobaric hypoxia has been summarized in Fig. 7.

## Conclusion

Aqueous extract of *Ganoderma lucidum* exhibits hippocampus dependent memory enhancing potential. Extract facilitated rapid acclimatization by activating HVR thus, normalizing perturbed blood gas/chemistry and prevented bioenergetic losses. Presence of extract alleviated glucocorticoid and glutamate buildup, modulated neurotransmission and improved synaptic plasticity by stimulating CREB/p-CREB/BDNF expression via ERK1/ERK2 induction and reducing α-SYN and SYN1 expression. Extract administration maintained O_2_ homeostasis by induction of EPO and NRF2/HO1 pathway and reduced stress-induced neuronal inflammation via stabilization of HIF1α/NFĸB axis thus preventing neuronal damage and vasogenic cerebral edema. Overall findings demonstrate first evidences on molecular targets of aqueous extract of *Ganoderma lucidum* involved in neuroprotective mechanism thereby strongly supporting its utility as effective therapeutic drug to combat HH-induced cognitive losses.

## Materials and Methods

### Extract preparation and standardization

*Ganoderma lucidum* (Voucher specimen DIP-GL/2011) was acquired from Pithoragarh, Uttarakhand, India and was characterized by Dr. Mousin, an ethnobotanist at the Defence Institute of Bio Energy Research, Haldwani, India. Aqueous extract was prepared in accelerated solvent system (ASE-350) equipped with a solvent controller unit (Dionex Corporation, CA, USA). Extraction was performed at room temperature (RT) for 15 min extraction time (5 min/cycle × 3 cycles) in 66 ml extraction cells containing sample and diatomaceous earth. Intermittent rinsing was performed at 60% purge volume to prevent cross contamination between cycles. The double distilled water (Milli-Q, Millipore) as solvent was injected into cells under 130 psi compression pressure, 1500 psi cell pressure and filtrate was collected in solvent bottles and transferred to lyophilizer (Allied frost, India) under sterile conditions. The lyophilized dried extract was stored at 4 °C for the further studies. Extract was quantified for phenolic, polysaccharide and flavonoid content and RP-HPLC identified presence of coumarin and ganoderic acid A in GLAQ^19^.

### *In vivo* study

#### Ethics statement

The study was approved by Institutional Animal Ethical Committee (DIPAS/IAEC/2017/16), DIPAS (Defence Institute of Physiology and Allied Sciences), Defence Research and Development Organisation (DRDO), Ministry of Defence, Delhi, India, and all the experimental protocols were performed in accordance with its relevant guidelines and regulations.

### Animals and hypobaric hypoxia exposure

Male Sprague-Dawley rats weighing 210–230 g were obtained from institute’s animal facility. They were housed in rooms maintained at 25 ± 1 °C, 55% ± 5% humidity and 12:12 h light-dark cycle. Animals were kept in a bedding of rice husk in polypropylene cages with free access to rodent pellet feed and potable water *ad libitum*.

In total 80 rats were used for the studies, out of which 50 rats were randomly divided into five groups (n = 10/group) as follows; group 1- Control: Unexposed untreated rats (Distilled water via gavage, in normoxic conditions at sea level); group 2-HH: Untreated hypobaric hypoxia exposed rats (distilled water via gavage; without GLAQ); group 3- HH + GLAQ100 (100 mg/kg body wt.); group 4- HH + GLAQ200 (200 mg/kg body wt.) and group 5- HH + GLAQ400 (400 mg/kg body wt.). Dose selection was based on our previous studies where GLAQ administration improved physical performance in rats exposed to hypobaric hypoxia^18^. Briefly, rats were trained in Morris Water Maze (MWM) for a stipulated period of time and post training, group 2–5 animals were inducted to a simulated altitude of 7620 m (25,000 ft at 282 mm Hg) for 7 days in a decompression chamber, while group 1 served as normoxia control. Animals were brought down to normobaric pressure (15 min every day) for replenishment of food and water along with oral administration of GLAQ as intended for clinical purpose. Post exposure, memory parameters of group 1–5 were recorded in MWM test and samples were obtained for analysis of hematology, oxidative stress, neurotransmitter and H&E staining. Similarly, remaining 30 rats were randomized into three groups (n = 10/group) as follows; group 1- Control, group 2- HH and group 3- HH + GLAQ200 for bio-efficacy studies at optimized dose based on above mentioned study parameters and exposed to HH in decompression chamber except group 1. Post exposure, samples were obtained for analysis of blood gas/chemical variables, bio-energetic markers, glucocorticoids, protein expression and IHC.

### Behavioral study

Spatial learning ability and reference memory was assessed in MWM, a hippocampus-dependent memory task frequently used in rodents^74,75^. The maze consisted of circular pool (120 cm diameter × 50 cm height, filled with water at 25 ± 1 °C) and a transparent escape platform (Plexiglas square placed 1 cm beneath the water surface). The pool was conceptually divided into four equal quadrants (Fig. 2a-a, schematic representation of virtual MWM). Platform was at affixed location that can be discriminated by visual cues. An overhead camera and computer assisted the tracking system ANY MAZE software (Stoelting, USA) for recording purpose. Briefly, two day habituation allowed rats to swim freely and explore tank for 5 minutes. Each animal was then subjected to four trials per day from each quadrant for five consecutive days where it was gently guided to find the hidden platform via shortest path within a search time of 60 seconds. Escape latency i.e., time to reach the platform from diagonally opposite quadrant was used as a measure of spatial-learning ability. The probe trial was conducted on 6^th^ day whilst the platform was removed and time spent in platform zone was recorded as a measure of memory retention.

### Sample collection and processing

In first dose dependent study, all fifty experimental rats of group 1–5 were bled under light anesthesia after which blood was collected for hematological analysis and plasma was separated for biochemical studies (n = 10/group). Then six animals from each group 1–5 were sacrificed by cervical dislocation and hippocampi was excised^76^ at 4 °C in ice-cold 0.1 M PBS and immediately snap frozen in liquid nitrogen. These tissues were later used for neurotransmitter analysis (n = 6/group). Remaining four animals from each group 1–5 were anesthetized and perfused transcardially with ice-cold 0.1 M PBS and chilled 4% paraformaldehyde (PFA) for the brain fixation. The fixed brain was dehydrated in graded series of alcohol, cleared in xylene and embedded in paraffin wax, followed by taking multiple transverse coronal sections (3 mm) from each block on coated slides for H&E staining (n = 4/group).

For bioefficacy study at optimized dose, blood was similarly collected from all thirty animals for blood gas/chemical analysis and plasma was separated for biochemical studies (n = 10/group). Hippocampus was collected from eighteen rats and homogenized in appropriate buffers (10% w/v) for analysis of bio-energetic markers, glucocorticoids, and protein expression (n = 6/group). Remaining twelve rats were perfused with ice-cold 0.1 M PBS and 4% PFA for the brain fixation. The fixed brain was thereafter preserved in glucose gradient (10%-20%-30%) at 4 °C. Next, multiple transverse sections of 20 mm and 10 mm thickness were cut from OCT embedded frozen brain on a sliding cryotome for IHC and CV staining, respectively (n = 4/group).

### Hematology and blood-gas/chemistry

Semi-automated blood analyzer (MS4, USA) was used to analyze following hematological parameters: WBC, RBC, Hct and Hb. Blood gas parameters such as pH, pCO_2_, pO_2_, BE, HCO_3_, TCO_2_, sO_2_ and lac were measured using i-STAT CG4+ cartridge (Abbott, USA catalog no. 03P85-25). The blood electrolyte and metabolite parameters: Na, K, Cl, iCa, glucose, BUN, creatinine, and AnGap were measured using i-STAT CHEM8+ cartridge (Abbott, USA catalog no. 09P31-25).

### Neurotransmitter analysis

Biogenic amines were quantified using Waters HPLC (Waters Corp, USA) equipped with W600 pump, 717 plus autosampler and 2465 electrochemical detector. Hippocampus tissues (100.0 mg + 2.21) homogenized in ice cold 0.05 M perchloric acid were centrifuged @10,000 rpm, 10 mins, 4 °C and supernatant was filtered with 0.22 µm membrane 13 mm syringe driven filter. The standards and samples were analyzed by maintaining the isocratic flow rate (1 ml/min) of the mobile phase (8.65 mM heptane sulphonic acid, 0.27 mM EDTA, 13% acetonitrile, 0.45% triethylamine, 0.25% phosphoric acid) using 3.9 × 300 mm C_18_RP PicoTag column. Detection was achieved using electrochemical detector set to +0.65 V potential and 50 nA current. Within the detection limits of analyzed catecholamines such as NE, E, DA, 5-HT and 5-HIAA (Sigma Aldrich, USA), working standard solutions (3–300 ng/ml) were prepared after dilutions from respective 1 mg/ml stock solutions. The levels of glutamate, belonging to class of excitatory neurotransmitters, were quantified in plasma and hippocampus lysate using commercial kit (BioAssay, USA). Briefly, kit was based on glutamate dehydrogenase catalyzed oxidation of glutamate thus forming colored product which was spectrophotometrically measured at 565 nm in multimode plate reader (BioTek, USA).

### Biochemical analysis

Oxidative stress was measured with estimations of MDA and GSH as per methods described elsewhere^77,78^. TAC was measured using commercial kit (BioAssay, USA) based on reduction of Cu^2+^ to Cu^+^ by antioxidant which forms colored product with a dye reagent and measured at 570 nm. Bioenergetic markers were measured using commercially available kits for measuring intracellular levels of ATP, NAD and NADH (BioAssay, USA). Briefly, ATP working reagent, provided in ATP assay kit, was added to lyse cells to release ATP which in the presence of luciferase reacts with the substrate D-luciferin to produce light which was spectrophotometrically measured. NAD/NADH kit was based on lactate dehydrogenase cycling reaction in which the NADH formed reduces a formazan reagent and the intensity of reduced product was measured at 565 nm. Stress response was measured with estimation of glucocorticoids: cortisol and corticosterone using commercial kits (Arbor DetectX, USA) provided with antibody coated microtiter plates based on ELISA principle. The generated colored product was measured at 450 nm after which online tool from MyAssays was used to calculate data as per manual instructions. Spectrophotometric readings were taken in multimode plate reader (BioTek, USA).

### Protein quantification

Commercially available ELISA kits from SinoGeneClon Biotech were used to quantify following protein markers in plasma and hippocampus lysate: NFĸB, HIF1α, HIF2α, VEGF, EPO, NRF2, HO1, BDNF, CREB, p-CREB, ERK 1/ERK 2, SYN 1 and α SYN. Protein levels were estimated in both plasma as well as hippocampus lysate (10% w/v in 0.1 M PBS) using Bradford reagent assay (Himedia, India). Equal amount of protein was then loaded into required wells of commercially available ELISA kits. Each kit was standardized as per manual instructions using bank control and serially diluting the standard to obtain standard curve for estimating unknown protein concentration. Spectrophotometric readings were taken in multimode plate reader (BioTek, USA).

### Histopathology and immunohistochemistry

Sections were stained with H&E^79^ for gross pathology and 0.1% CV dye^80^ for morphology of neurons. In CV stain, the number of densely stained irregular shaped pyknotic neurons was counted at 200 µm scale bar with a 40X objective (total magnification 400x) using LMI-BM500 microscope (LMI, UK). Two random sections per sample were observed and randomly counted in triplicates by an observer blinded to the treatment conditions. Neuronal damage was calculated as percentage neuronal damage (number of damaged (pyknotic) cells/total no of cells × 100) per counting field. For IHC, cryosections were gently washed with 0.1 M PBS and then incubated in sodium citrate tribasic for 10 min at 95 °C for antigen retrieval. 3% H_2_O_2_ was used for peroxidase block followed by washing in 0.1 M PBS. Blocking was done in 1% BSA in 0.1% PBST (triton-x in 0.1 M PBS) for an hour at 4 °C. Sections were then incubated overnight at 4 °C in 1:100 primary antibodies: anti-HIF1α (Sigma Aldrich, USA), anti-BDNF (ThermoFisher Scientific, USA) and anti-SYN 1 (ThermoFisher Scientific, USA). After washing, sections were then incubated accordingly in 1:200 HRP conjugated secondary antibodies (Abcam, UK) of anti-mouse or anti-rabbit for 2 hr at 4 °C, followed by washing in 0.1 M PBS and staining in DAB. After mounting, slides were observed under light microscope for qualitative analysis.

### *In vitro* study

#### Cells and hypoxia exposure

HT22, subclone of the HT4 murine hippocampal cells (kind gift of Dr. Dave Schubert, Salk Institute, San Diego, CA, USA) were maintained as described elsewhere^55^. Cells plated at a density of 1 × 10^4^ cells/ml in 96 well plates were allowed to adhere for 24 h. Next, untreated unexposed cells (control) were cultured under normoxic (21% O_2_) conditions in an incubator (Heracell 150i, Thermo Scientific, USA) maintaining 5% CO_2_, 37 °C while, GLAQ (0, 25, 50, 100 µg/ml) treated cells were exposed to hypoxia (0.5% O_2_) for 24 hr in hypoxia chamber (New Brunswick Galaxy 48 R) maintained at 37 °C, 5% CO_2_, 94.5% N_2_ atmosphere. Spectrophotometric readings were taken in multimode plate reader (BioTek, USA).

### Cell viability assay

50 µl of 1 mg/ml of 3-(4,5-dimethylthiazol-2-yl)-2,5-diphenyl tetrazolium bromide (MTT) dye (Sigma Aldrich, USA) in 1X phosphate buffer saline (PBS) was added to each well and incubated for 3 h in normoxic conditions. Formazan crystals were then solubilized in 100 µl of dimethyl sulfoxide by incubating in shaking condition at RT for 5 minutes. Absorbance was recorded at 570 nm using 630 nm as reference filter. Absorbance of control was taken as 100% cell survival.

### Estimation of redox status

Intracellular ROS was measured using the fluorescent dye 2, 7- dichlorofluorescein diacetate (DCFH-DA; Sigma Aldrich, USA). Briefly, cells were washed with 1X PBS followed by incubation in 100 µl of 10 µM DCFH-DA in 1X PBS for 30 min at 37 °C. Fluorescence was detected with excitation and emission at 485 nm 530 nm filter, respectively. Percentage change was calculated over 100% control.

### Cignal finder array

High throughput 10-pathway Cignal Finder Stress and Toxicity reporter array catalog no. Qiagen CCA-107L (Qiagen, Germany) based on principle of reverse transfection was used for cell-based multi-pathway activity assay. Experiments were performed according to instruction in manual provided with kit. Post exposure, Dual-Glo luciferase assay system catalog no. E2920 (Promega, USA) was used to measure firefly luciferase and renilla luciferase activity. Readings were normalized with respect to control according to manual instructions. Data has been represented as fold change of a particular gene over basal control value.

### Statistical analysis

The results are expressed as mean ± standard error mean. The data was subjected to one-way ANOVA tests followed by Bonferroni test for multiple inter-group variations (Graph Pad InSTAT 3). p-value less than 0.05 was considered to be statistically significant where ‘*’ denotes comparison with control group and ‘^#^’ denotes comparison with untreated stress exposed group.

## Supplementary information

Supplementary Information.
