# Supplementary material for: Ganoderma lucidum aqueous extract prevents hypobaric hypoxia induced memory deficit by modulating neurotransmission, neuroplasticity and maintaining redox homeostasis
Source: Sci Rep. 2020 Jun 2;10:8944. doi: 10.1038/s41598-020-65812-5 (PMC7265456; doi:10.1038/s41598-020-65812-5)
Supplement: Supplementary file 1 — Supplementary Information. [file 41598_2020_65812_MOESM1_ESM.pdf]

Ganoderma lucidum aqueous extract prevents hypobaric hypoxia induced memory deficit by modulating neurotransmission, neuroplasticity and maintaining redox homeostasis

Purva Sharma, Rajkumar Tulsawani*

Defence Institute of Physiology and Allied Sciences (DIPAS),

Lucknow Road, Timarpur, Delhi-110 054, India.

*Correspondence: Dr. Rajkumar Tulsawani

Defence Institute of Physiology and Allied Sciences

Lucknow Road, Timarpur, Delhi – 110 054, India;

Tel: +91-11-23883304, Fax: +91-11-23914790;

E-mail: rktulsawani@yahoo.com

TS1. Effects of GLAQ in presence and absence of hypobaric hypoxia stress on memory impairment and pathological markers in male SD rats in presence and absence of hypoxia.

**PARAMETERS**	**NORMOXIA**	**NORMOXIA + GLAQ 100**	**HH**	**HH + GLAQ 100**	**p-value**
Escape latency (seconds)	5.28 ± 0.61	5.93 ± 0.93	17.46 ± 1.70*	10.74 ± 1.24	0.0001
Time spent in platform zone (seconds)	26.42 ± 2.23	23.92 ± 1.93	17.84 ± 1.51*	22.34 ± 1.62	0.0308
Nor epinephrine (fold change)	1.10 ± 0.01	1.09 ± 0.21	1.82 ± 0.21*	1.14 ± 0.10	0.024
5-hydroxytryptamine (fold change)	1.01 ± 0.11	1.06 ± 0.09	0.61 ± 0.09*	0.76 ± 0.08	0.0265
ALT (U/I)	17.0 ± 0.99	19.5 ± 1.10	30.4 ± 1.44**	24.8 ± 2.54*	0.0001

- Data expressed as Mean ± SEM for n=5 with One way ANOVA : Dunnett's Test for comparison with normoxia.
  HH, hypobaric hypoxia.
- In absence of stress under normoxic conditions, GLAQ 100 mg/kg showed no effect on spatial memory (escape latency, time spent) whereas under HH stress, GLAQ improved memory in exposed treated rats. Similar effects were obtained in levels of NE, 5-HT and ALT (alanine transaminase).

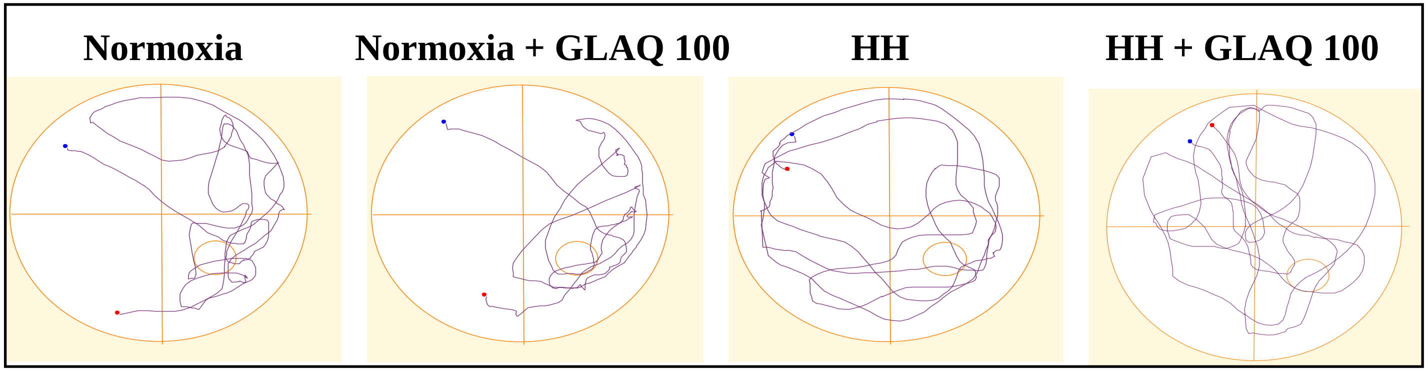

FS1. **Effects of GLAQ on spatial memory of male SD rats in presence and absence of hypobaric hypoxia (HH).** Representative track plot of probe trial in Morris water maze test of control and HH exposed rats with and without GLAQ 100 mg/kg indicates protective effects during hypoxia whereas, absence of *per se* effects under normoxia.
